# Challenges and strategies in adolescent vaccination: Number 12 – 2024

**DOI:** 10.61622/rbgo/2024FPS12

**Published:** 2024-11-14

**Authors:** Cecília Maria Roteli-Martins, Renato de Ávila Kfouri, André Luís Ferreira Santos

**Affiliations:** Faculdade de Medicina do ABC Santo André SP Brazil Faculdade de Medicina do ABC, Santo André, SP, Brazil.; Sociedade Brasileira de Pediatria Brazil Sociedade Brasileira de Pediatria; Universidade de Taubaté Brazil Universidade de Taubaté

## Key points

- Over the last 10 years, the immunological protection of vaccines administered in the first years of life has progressively decreased, and this is associated with a higher than expected incidence of vaccine-preventable diseases in adolescents and young adults.
- The greater circulation of pathogens among adolescents and young adults leads to an elevated risk of infection in unvaccinated or not fully vaccinated younger children.
- Vaccination of adolescents is important to increase the benefits of immunization more equitably beyond childhood.
- Even though after the COVID-19 pandemic, several programs were launched to increase the use of different vaccines in adolescent populations around the world, vaccination coverage (VC) among adolescents remains suboptimal.
- It is very important that gynecologists are aware of the challenges in effectively vaccinating adolescents against the main vaccine-preventable diseases affecting this age group.
- Sharing knowledge of the evidence on strategies that can be adopted to improve adherence to immunization in adolescents is of great interest, especially during a gynecological consultation.

## Recommendations

- Febrasgo understands the negative impact of vaccine-preventable infections on adolescents and recommends that health professionals be aware of opportunities to approach vaccination during routine consultations with adolescents and their responsible family members.
- Febrasgo has the role of bringing knowledge of evidence-based studies on vaccines available in adolescence and the need for boosters for an effective protection as a shared decision between professionals and their patients.
- Febrasgo understands the knowledge gap around interventions to improve vaccine adherence among adolescents and recommends adopting the strategy of vaccination in schools to improve VC among adolescents with better adherence to current and future vaccines.
- Febrasgo defends the strengthening of vaccination of adolescents in the National Immunization Program and the inclusion of new vaccines, such as those against meningococcal disease serogroups, in addition to serogroup C.
- The Febrasgo National Specialized Commission on Vaccines understands the need to take advantage of every opportunity to inform and update health professionals about the benefits and risks of vaccines and vaccine-preventable diseases, so that they can make the necessary choices and recommendations for their adolescent patients, thereby reducing deaths, hospitalizations and negative impacts on families and health systems.
- With the necessary and correct information, Febrasgo understands it may be possible to reduce vaccine hesitancy and reduce morbidity and mortality related to pathogens, especially those affecting adolescents, such as the various meningococcal serogroups that cause meningococcal disease.

## Background

In 2021, approximately 1,500,000 adolescents and young adults aged 10-24 years died, that is, 4,500 deaths per day. The survival chances of adolescents and young adults vary greatly around the world. In 2021, the probability of dying among people aged 10-24 years was higher in Sub-Saharan Africa and lower in Europe and North America. The global average probability of a 10-year-old child dying before the age of 24 years was about six times greater in Sub-Saharan Africa than in North America and Europe.^(1)^

It is important to highlight that mortality patterns in people aged 10-24 years reflect the underlying risk profiles of the age groups, and those of young people aged 10-14 years are dominated by infectious diseases, including vaccine-preventable diseases.^(2)^

The World Health Organization (WHO) recommends several vaccines for children aged 10-19 years, and some of these vaccines are offered mainly to this age group, such as the human papillomavirus (HPV) vaccine. Others are booster vaccines, also administered to younger children, such as vaccines against hepatitis B, diphtheria, tetanus and pertussis.^(2)^

Many adolescents do not receive recommended vaccines, and different approaches have been tested to change this situation. One approach is to involve not only adolescents, but also their parents and communities by providing them with information about vaccines or even reminding them of vaccines expiration dates and the benefits of vaccination. Another approach is to involve health professionals by sharing the knowledge and benefits of immunization through educational activities. A third approach is to make vaccines more accessible to people by making them available for free or offering them closer to home with flexible schedules, including at schools. A fourth approach is to pass vaccination laws, such as in some countries, where students must prove they have been vaccinated before they can attend school.^(3)^

In the spite of low and declining incidence trends of Invasive Meningococcal Disease (IMD) in recent decades, this disease causes panic among the population, mainly due to its potential epidemic nature, rapid onset, high fatality rates (10%–20%), morbidity, and risk of complications (up to 20% of MD survivors may develop long-term sequelae, including neurological deficits, ocular and hearing impairment, or limb amputation). [Stephens DS, Greenwood B, Brandtzaeg P. Epidemic meningitis, meningococcaemia, and Neisseria meningitidis. Lancet. 2007;369 (9580):2196–1210. doi:10.1016/S0140-6736(07)61016-2.]

The causative agent of MD, Neisseria meningitidis, is a gram-negative, aerobic, nonmotile diplococcus belonging to the Neisseriaceae family. The antigenic composition of the polysaccharide capsule allows the classification of Neisseria meningitidis into 12 different serogroups: A, B, C, H, I, K, L, W, X, Y, Z, and E. Currently, serogroups A, B, C, Y, W, and X are responsible for almost all cases of the disease, infecting only humans.

The prevalence of meningococcus serotype B in Brazil has varied over time. Serogroup B was a significant cause of meningococcal disease during the 1990s and early 2000s. Between 1990 and 2001, serogroup B accounted for 67% of invasive Neisseria meningitidis isolates, and between 1997 and 1998, approximately 60% of laboratory-confirmed meningococcal disease cases were identified as serogroup B. [Chicuto LAD, de Moraes C, Cássio de Moraes J, Sáfadi MAP. A Critical Analysis of Serogroup B Meningococcal Disease Burden in Brazil (2001-2015): Implications for Public Health Decisions. Human Vaccines & Immunotherapeutics. 2020;16(8):1945-1950. doi:10.1080/21645515.2019.1700710.]

However, there has been a shift in serogroup prevalence over the years. From 2002 to 2017, serogroup B accounted for 38.1% of isolates with a remarkable decrease in prevalence as serogroup C became more dominant. In specific regions such as Rio Grande do Sul, serogroup B accounted for 69.8% of cases from 1995 to 2003. [Chicuto LAD, de Moraes C, Cássio de Moraes J, Sáfadi MAP. A Critical Analysis of Serogroup B Meningococcal Disease Burden in Brazil (2001-2015): Implications for Public Health Decisions. Human Vaccines & Immunotherapeutics. 2020;16(8):1945-1950. doi:10.1080/21645515.2019.1700710.]

Vaccination coverage against bacterial meningitis at levels close to 90% provides a marked reduction in the number of cases of meningococcal disease among those vaccinated, and this was evidenced after the introduction of the MenC vaccine (the first vaccine approved against meningococcus) into public programs in several countries around the world.^(4)^ Countries that included adolescents in their vaccination programs obtained significant results in indirect protection, since most asymptomatic carriers of the bacteria are concentrated in this age group.^(5)^

The vaccines currently available to prevent meningococcal disease are those that contain capsular polysaccharides from meningococcal serogroups A, C, W and Y conjugated to different carrier proteins, depending on the manufacturer. These expand the spectrum of protection in relation to the monovalent C vaccine and have been recommended by scientific societies and Febrasgo in the calendar for children and adolescents. The Ministry of Health has made the ACWY vaccine available in the Unified Health System (SUS) in a single dose schedule for ages 11-14 years on a temporary basis, and for other age groups only in the private network.^(6,7)^

Early adolescence (9-14 years) is the ideal time for vaccination against HPV infection and it is estimated that if 90% of adolescent girls worldwide receive the HPV vaccine, more than 40 million lives could be saved in the next century. However, it is estimated that in 2021, only 12% of these adolescents were vaccinated.^(8)^

In 2020, the Cochrane Database of Systematic Reviews published a review with the aim of evaluating the effects of interventions to improve vaccination adherence among adolescents. Several strategies to improve adolescent vaccination were evaluated in this review, including health education, financial incentives, mandatory vaccination, and vaccine distribution in schools. As most of the evidence was of low to moderate level, although the review indicates some of the likely effects of these interventions, the chance of the effects being substantially different is high, and additional studies are needed to further improve adolescent immunization strategies, especially in low- and middle-income countries, where vaccination programs for this age group are limited. Furthermore, the authors concluded it is essential to understand the factors influencing hesitancy, acceptance and demand for vaccination among adolescents in different contexts.^(3)^

Vaccine adherence during adolescence is a global issue influenced by multiple factors, such as the lack of programmatic preventive measures, health consultations at this age, weak medical advice on the importance of vaccination, and the lack of school entry requirements for vaccination of adolescents. Therefore, the role of health professionals, including gynecologists, is crucial in recommending vaccination for adolescents, taking advantage of consultation opportunities or even vaccination campaigns in schools.

## What are the main vaccines and the benefits of vaccinating during adolescence?

Meningococcal B and quadrivalent conjugate vaccines (types A, C, W and Y) should be considered the best options for immunizing adolescents against meningococcal disease. Febrasgo recommends immunization with the single dose ACWY vaccine and two doses of meningococcal B at a one-month interval in between. The unpredictability of meningococcal disease associated with the asymptomatic carrier state of young people, habits and behaviors that facilitate its spread, such as traveling and commuting, together with the epidemic potential of the bacteria makes the use of multivalent vaccines extremely desirable, as it broadens the spectrum of protection against the disease.^(7)^

Targeting adolescents for vaccination immediately generates three benefits: catching up on missed vaccinations, boosting waning immunity, and achieving primary immunization with new vaccines.^(10)^ Vaccines administered during adolescence include, but are not limited to those against HPV, diphtheria, tetanus, whooping cough, measles, mumps, rubella, chickenpox, hepatitis B, polio, meningococcal disease and, currently, the vaccine against COVID-19.^(11)^

## What is the main challenge in approaching vaccination during a routine appointment with an adolescent?

As in many places, adolescents often seek physicians only when they are sick and not as a routine prevention, the main challenge is the limited opportunity to inform them about the importance of vaccines and that they should be administered. In these cases, adolescents may be more interested in their current health status than in the possible benefits of preventing future vaccine-preventable diseases.^(12)^ Although schools have been widely used as distribution platforms for vaccinating a large number of school-age children,^(13)^ school-based vaccination programs may not be fully successful in countries with suboptimal school attendance rates, such as many low- and middle-income countries, where these rates vary because of several factors; geographic location, socioeconomic profile and also gender issues.^(14)^ Strategies such as mass immunization campaigns can be used to complement school vaccination programs in places with low school attendance rates.^(11)^

## What are the VC rates among adolescents in the world and in Brazil?

Data on VC among adolescents are limited, but VC is generally low in this group.^(15)^ Across Brazil, initial meningococcal vaccine coverage was high, exceeding 95% for infants, but it declined over time, reaching 83% in 2018. However, vaccination uptake among adolescents was notably lower, with less than 20% coverage in 2017–2018. The COVID-19 pandemic further exacerbated the decline in vaccination rates, with a reported 10–20% reduction in overall vaccination coverage during 2019–2020. Overall, although the introduction of meningococcal vaccines has led to a significant reduction in the incidence and mortality of meningococcal disease, particularly for serogroup C, maintaining high coverage rates remains a challenge, especially among adolescents and in the context of the COVID-19 pandemic. [Aparecido Nunes A, De Jesus Lopes De Abreu A, Cintra O, et al. Meningococcal Disease Epidemiology in Brazil (2005-2018) and Impact of MenC Vaccination. Vaccine. 2021;39(3):605-616. doi:10.1016/j.vaccine.2020.11.067]. [Silveira MM, Conrad NL, Leivas Leite FP. Effect of COVID-19 on Vaccination Coverage in Brazil. Journal of Medical Microbiology. 2021;70(11). doi:10.1099/jmm.0.001466.]

HPV vaccination coverage rates are also considered low, with an estimated 6.1% of adolescents worldwide having completed the HPV vaccination series in 2014, with a large variation between low- and middle-income countries, and high-income countries. Human Papillomavirus vaccination coverage was just 1.1% in Asia and 1.2% in Africa, compared to 35.6% in North America and 35.9% in Oceania.^(8)^ In Brazil, in 2023, HPV vaccination coverage was 70% with the two recommended doses accumulated for girls, not reaching the target of 90% recommended by the WHO.^(16)^ Globally, the average of HPV programs achieves VC of 55% for the first dose and 44% for the last dose of the HPV vaccine, and the HPV VC has reduced by less than 15% since 2019, reflecting a downward trend that continued in 2021. This low coverage combined with the large population without access to HPV vaccines results in a very low global coverage (12%).^(1)^

## What are the main barriers to vaccinating adolescents?

Adolescence is a particular period of life characterized by changes in intellectual, moral, physical, emotional and psychological development. All of this can have a considerable impact on compliance with vaccination schedules, because the approach to any preventive method no longer depends entirely on the judgment of parents and pediatricians, as in the first years of life, but is the consequence of a more complex process involving adolescents, their thoughts and opinions, the relationship with their parents, friends and physicians, and the information they receive from mass media.^(12)^ The most commonly reported barriers to vaccinating adolescents include: lack of knowledge about vaccines; negative attitudes towards vaccination on the part of adolescents, parents, teachers and health professionals; poor vaccine infrastructure; and financial constraints.^(17)^

## What strategies can be implemented to increase VC rates in adolescence?

Interventions to increase adolescents' adherence to vaccination can have multiple components, targeting adolescents and their communities, health professionals, the health system, or a combination of these.^(3)^ A strategy is to involve relevant government bodies in the planning, implementation and monitoring of the National Immunization Program with vaccination moving from health centers to schools, ensuring a complete series of vaccinations for adolescents.^(3)^ In addition to choosing schools as the primary vaccination site, a strategy involving vaccination on the same day can be adopted, that is, the consultation, prescription and administration occurring at the same place.^(18)^ Other components cited as strategies involve sending messages informing adolescents about vaccinations that are due or have already expired and monitoring program performance to ensure effective, socially fair and cost-effective immunization results.^(19)^

## Are there educational interventions to increase vaccination adherence targeting adolescents?

Educational interventions allow adolescents and their communities to understand the meaning and relevance of vaccination for their health (Willis et al., 2013; Kaufman et al., 2017).^(20,21)^ Such interventions can be made in person or through messages, social media conversations, audiovisual presentation or role-playing, printed materials, websites, multimedia campaigns, or community events.^(3)^ These types of interventions can be targeted at individuals or groups and include information about health and nutrition; the risks and benefits of vaccines; where, how and when to access vaccine services; who should be vaccinated; or a combination of them. Adolescents and communities can receive vaccine education through posters displayed in waiting rooms, brochures, emails, and website resources.^(22)^

## What is the role of the gynecologist in increasing vaccination adherence in adolescence?

Gynecologists must clearly clarify and advise parents and adolescents about vaccination and mention its benefits in preventing disease and possible adverse events. It is essential that gynecologists stay up to date about vaccine knowledge, especially when new vaccines are recommended.^(7,12)^ Careful and truthful counseling about vaccination of adolescents and their parents by healthcare professionals may result in adolescents’ greater willingness to be vaccinated. Health system interventions ensure that vaccines are available when adolescents and their communities are able to demand from government agencies.^(23)^

## Final considerations

Several factors may play a role in reducing vaccination coverage among adolescents, but some challenges are substantially the same as barriers to immunization of children and adults. However, as adolescents are personally involved in decision-making, organizing vaccine administration and whether or not to accept vaccination is more complicated and there is a greater risk of missed opportunities. Experts on the subject agree that one of the strategies to increase vaccination coverage among adolescents is clear information from health professionals for adolescents and their families. Immunizing adolescents against meningococcal disease and HPV vaccination are important extensions of the traditional immunization schedule, which has long been aimed almost exclusively at children. The National Specialized Commission on Vaccines of Febrasgo, along with this Febrasgo Position Statement, aims to show these challenges and strategies to assist in a new preventive approach that can have positive effects not only on adolescents themselves, but also on the community.

## National Specialized Commission on Vaccines of the Brazilian Federation of Gynecology and Obstetrics Associations (FEBRASGO)

**President:**

Cecilia Maria Roteli Martins

**Vice-president:**

Nilma Antas Neves

**Secretary:**

Susana Cristina Aidé Viviani Fialho

**Members:**

André Luis Ferreira Santos
Angelina Farias Maia
Fabiola Zoppas Fridman
Giuliane Jesus Lajos
Isabella de Assis Martins Ballalai
Juarez Cunha
Julio Cesar Teixeira
Manoel Afonso Guimaraes Goncalves
Marcia Marly Winck Yamamoto De Medeiros
Renata Robial
Renato de Ávila Kfouri
Valentino Antonio Magno

## References

[1] World Health Organization (2017). Reducing Missed Opportunities for Vaccination (MOV).

[2] World Health Organization (2023). Adolescent and young adult health.

[3] Abdullahi LH, Kagina BM, Ndze VN, Hussey GD, Wiysonge CS (2020). Improving vaccination uptake among adolescents. Cochrane Database Syst Rev.

[4] Ministério da Saúde (2021). Situação epidemiológica.

[5] Hamborsky J, Kroger A, Wolfe C, Centers for Disease Control and Prevention (2015). Epidemiology and prevention of vaccine-preventable diseases.

[6] Ministério da Saúde (2023). Calendário de vacinação: adulto e idoso.

[7] Roteli-Martins CM, Neves NA, Magno V, Kfouri R (2022). Vacinação para doença meningocócica. Femina.

[8] Bruni L, Saura-Lázaro A, Montoliu A, Brotons M, Alemany L, Diallo MS (2021). HPV vaccination introduction worldwide and WHO and UNICEF estimates of national HPV immunization coverage 2010-2019. Prev Med.

[9] World Health Organization (2024). Human papillomavirus and cervical cancer.

[10] Brabin L, Greenberg DP, Hessel L, Hyer R, Ivanoff B, Van Damme P (2008). Current issues in adolescent immunization. Vaccine.

[11] Piot P, Larson HJ, O'Brien KL, N'kengasong J, Ng E, Sow S (2019). Immunization: vital progress, unfinished agenda. Nature.

[12] Principi N, Esposito S (2013). Adolescents and vaccines in the western world. Vaccine.

[13] Cawley J, Hull HF, Rousculp MD (2010). Strategies for implementing school-located influenza vaccination of children: a systematic literature review. J Sch Health.

[14] Mackroth MS, Irwin K, Vandelaer J, Hombach J, Eckert LO (2010). Immunizing school-age children and adolescents: experience from low- and middle-income countries. Vaccine.

[15] Loke AY, Kwan ML, Wong YT, Wong AK (2017). The uptake of human papillomavirus vaccination and its associated factors among adolescents: a systematic review. J Prim Care Community Health.

[16] Ministério da Saúde Título do documento. Ano?.

[17] Ngcobo NJ, Burnett RJ, Cooper S, Wiysonge CS (2018). Human papillomavirus vaccination acceptance and hesitancy in South Africa: research and policy agenda. S Afr Med J.

[18] Bednarczyk RA, Brewer NT, Gilkey MB, Zorn S, Perkins RB, Oliver K (2023). Human papillomavirus vaccination at the first opportunity: an overview. Hum Vaccin Immunother.

[19] Bigaard J, Franceschi S (2021). Vaccination against HPV: boosting coverage and tackling misinformation. Mol Oncol.

[20] Stinchfield PK (2008). Practice-proven interventions to increase vaccination rates and broaden the immunization season. Am J Med.

[21] Willis N, Hill S, Kaufman J, Lewin S, Kis-Rigo J, De Castro Freire SB (2013). "Communicate to vaccinate": the development of a taxonomy of communication interventions to improve routine childhood vaccination. BMC Int Health Hum Rights.

[22] Kaufman J, Ames H, Bosch-Capblanch X, Cartier Y, Cliff J, Glenton C (2017). The comprehensive ‘Communicate to Vaccinate’ taxonomy of communication interventions for childhood vaccination in routine and campaign contexts. BMC Public Health.

[23] Kaddar M, Schmitt S, Makinen M, Milstien J (2013). Global support for new vaccine implementation in middle-income countries. Vaccine.

